# Epigenetic disruption of adipogenic gene enhancers in dedifferentiated liposarcomas and its therapeutic value

**DOI:** 10.3389/fonc.2025.1419877

**Published:** 2025-04-30

**Authors:** Naoko Hattori, Hironori Takamatsu, Naoko Iida, Naofumi Asano, Satoshi Yamashita, Gina Miku Oba, Kozue Kimura, Akihiko Yoshida, Eisuke Kobayashi, Robert Nakayama, Morio Matsumoto, Masaya Nakamura, Akira Kawai, Toshikazu Ushijima

**Affiliations:** ^1^ Department of Epigenomics, Institute for Advanced Life Sciences, Hoshi University, Tokyo, Japan; ^2^ Division of Epigenomics, National Cancer Center Research Institute, Tokyo, Japan; ^3^ Laboratory of Integrative Metabolic Regulation, Institute for Molecular and Cellular Regulation, Gunma University, Maebashi, Japan; ^4^ Department of Orthopaedic Surgery, Keio University School of Medicine, Tokyo, Japan; ^5^ Department of Life Engineering, Faculty of Engineering, Maebashi Institute of Technology, Maebashi, Japan; ^6^ Laboratory of Computational Genomics, Institute for Quantitative Biosciences, The University of Tokyo, Tokyo, Japan; ^7^ Department of Diagnostic Pathology, National Cancer Center Hospital, Tokyo, Japan; ^8^ Department of Musculoskeletal Oncology, National Cancer Center Hospital, Tokyo, Japan

**Keywords:** epigenome, DNA methylation, sarcoma, adipogenic differentiation, DNA demethylating agent

## Abstract

Liposarcoma (LPS) is the most common soft-tissue sarcoma in adults, and well-differentiated liposarcoma (WDLPS) and dedifferentiated liposarcoma (DDLPS) are the most frequent subtypes. These LPSs are considered to develop due to disturbances in the adipogenic differentiation of mesenchymal stem cells. However, the molecular mechanisms underlying the disturbances remain unclear. Here, we aimed to identify the mechanism and explore its therapeutic advantages focusing upon their epigenetic alterations, known to be important in differentiation. First, we conducted a genome-wide DNA methylation analysis using 15 LPSs (6 WDLPSs and 9 DDLPSs) and 6 normal adipose tissues. Unsupervised hierarchical cluster analysis using DNA methylation profiles at enhancers classified the samples into the three histological types, whereas analysis using promoters did not. Principal component analysis revealed that normal adipose tissues and WDLPSs were grouped closely, whereas DDLPSs were scattered. Genomic regions hypermethylated in DDLPSs were enriched for enhancers, especially super-enhancers (13.5% of hypermethylated regions and 7.0% of the whole genome), which were located in the genes involved in adipogenesis, such as *PPARG2* and its target genes (*FABP4* and *PLIN1*). In addition, marked decreases in *PPARG2* and *FABP4* expression were confirmed in DDLPSs. Then, treatment of *PPARG2*-expressing DDLPS cell lines with 5-aza-2’-deoxycytidine, a DNA demethylating agent, and rosiglitazone, a PPARγ agonist, was shown to induce differentiation with enhanced expression of *FABP4*. These findings indicate that aberrant DNA methylation of adipogenic gene enhancers plays a crucial role in the development of DDLPS and can be a therapeutic target.

## Introduction

Liposarcoma (LPS) is one of the most common adult soft-tissue sarcomas, and well-differentiated liposarcomas (WDLPS) and dedifferentiated liposarcomas (DDLPS) constitute the most frequent subtypes (48 - 58% of all LPS) (1, 2). Retroperitoneal DDLPSs have metastatic potential and showed a 6-fold increased risk of death compared to retroperitoneal WDLPSs (3, 4). Approximately 10% of DDLPS cases arise from WDLPS, and WDLPS and DDLPS share similar characteristics of genetic aberrations (5–10). Both WDLPS and DDLPS are considered to develop because of disturbances in normal adipogenic differentiation from mesenchymal stem cells (11). Among DDLPS, those in the retroperitoneum are known to have a worse prognosis than those in the extremities (12).

The involvement of epigenetic alterations has been indicated as a mechanism of disturbance in differentiation. Among multiple types of sarcomas, DNA methylation profiles successfully distinguished multiple types of sarcomas (13), and DDLPS from other types of sarcomas (14). Within LPSs, methylation-silencing of specific genes, such as *CEBPA* and *miR-193b*, was characteristic of DDLPSs (15). Even when limited to DDLPSs, DNA methylation profiles produced two groups with distinct leukocyte infiltration and disease-specific survival (16). However, despite the recent recognition of the essential role of enhancers in determining cell fate (17, 18), information on DNA methylation alterations in enhancer regions remains limited because the microarrays used in early studies had a limited number of probes in enhancer regions. Noteworthily, enhancers are particularly sensitive to aberrant DNA methylation (19), and their methylation alterations are expected to be important in disturbances in differentiation.

In this study, we aimed to elucidate the role of DNA methylation alterations in the enhancers of DDLPS development and explore the therapeutic value of targeting epigenetic disruption in DDLPS.

## Materials and methods

### Liposarcomas and normal adipose tissues

Frozen samples of 6 WDLPSs, 9 DDLPSs, and 6 normal adipose tissues were obtained from the National Cancer Center Biobank (Tokyo, Japan). Among them, 4 WDLPSs, 3 DDLPSs, and 6 normal adipose tissues were obtained from the extremities, and 2 WDLPSs and 6 DDLPSs were obtained from the retroperitoneum. The detailed clinical and pathological information is shown in **Supplementary Table S1**. For extraction of genomic DNA and total RNA, frozen normal adipose tissues and tumors were ground using a Multi-beads Shocker (Yasui Machine, Osaka, Japan). Genomic DNA was extracted using standard phenol-chloroform extraction and ethanol precipitation methods. Total RNA was extracted using ISOGEN reagent (Nippon Gene, Tokyo, Japan) and purified using the RNeasy MinElute Cleanup Kit (Qiagen, Hilden, Germany). The mean ages of the groups of WDLPS, DDLPS, and normal control were 53.5, 63.9, and 56.3 years old, respectively. The specimens were obtained with written informed consents from patients who underwent surgery. This study was approved by the Institutional Review Board of the National Cancer Center (protocol number 2004-050).

### Liposarcoma cell lines

Human WDLPS cell lines (93T449 and 94T778) and DDLPS cell lines (LP6 and LSP12) were a kind gift from Dr. Andrew J Wagner at Dana-Farber Cancer Institute (Boston, MA, USA). The cells were cultured in RPMI-1640 (FUJIFILM Wako Pure Chemical Corp., Osaka, Japan) supplemented with 10% fetal bovine serum and 1% penicillin/streptomycin at 37°C in a humidified atmosphere with 5% CO_2_. The cells were checked for Mycoplasma infection using the MycoAlert Mycoplasma Detection Kit (Lonza, Basel, Switzerland).

### Genome-wide DNA methylation analysis

Genome-wide DNA methylation analysis was performed using the Infinium MethylationEPIC BeadChip (Illumina, San Diego, CA) (v1.0 for normal adipose tissues, primary liposarcomas, and normal peripheral leukocytes, and v2.0 for DDLPS cell lines) as described previously (20, 21). Each sample was analyzed once because high analytical reproducibility is known for this microarray (22). The microarray assessed the degree of methylation of 865,860 probes (MethylationEPIC BeadChip v1.0) and 937,056 probes (MethylationEPIC BeadChip v2.0) with a β-value ranging from 0 (unmethylated) to 1 (fully methylated). For the data from MethylationEPIC BeadChip v1.0, to adjust probe design biases, β-mixture quantile (BMIQ) normalization was conducted using a web tool, MACON (23). To reduce the data size for convenience of handling, using MACON, the CpG probes were grouped into 538,616 genomic blocks (GBs) consisting of probes within 500 bp. The GBs were classified based on their locations relative to a transcription start site (TSS) [TSS200 (200 bp upstream region from TSS), TSS1500 (regions between 200 bp upstream and 1500 bp upstream from TSS), 5’-UTR, first exon, gene body, 3’-UTR, and intergenic region] and their locations against a CpG island (CGI) (N Shelf, N Shore, CGI, S Shore, S Shelf, and non-CGI). The annotation for MethylationEPIC originating from the ENCODE and FAMTOM5 projects was used to define enhancer regions. To address the issue of cellular heterogeneity, as in our previous studies (24, 25), tumor cell contents were estimated using DNA methylation statuses of 262 genomic blocks (GBs) unmethylated (β-value < 0.2) in normal peripheral leukocytes and normal adipose tissues but methylated (β-value > 0.8) in three or more of four liposarcoma cell lines (**Supplementary Figure S1A**). Enrichment of a binding motif compared to Refseq coding genes was conducted using ChIP-Atlas (https://chip-atlas.org).

### Quantitative reverse transcription PCR

Complementary DNA (cDNA) was synthesized from total RNA using oligo-(dT)_12-18_ (Thermo Fisher Scientific, Waltham, MA), SuperScript III Reverse Transcriptase for primary samples and LP6 cells, and SuperScript IV for LPS12 cells. Quantification of cDNA molecules was performed by RT-qPCR using specific primers shown in **Supplementary Table S1**, SYBR Green I (Lonza), and the CFX Connect Real-Time PCR Detection System (Bio-Rad, Hercules, CA). The number of cDNA molecules of a target gene in a sample was determined by comparing an amplification curve with those of standard DNA samples with known copy numbers. The quantified number of target cDNA molecules was normalized to that of *GAPDH* cDNA molecules.

### Overexpression of PPARγ2

pCMV-full-length *PPARG2* vector and pCMV-nuclear transported *EGFP* vector were purchased from VectorBuilder (Yokohama, Japan) and were introduced into LP6 and LPS12 cell lines using Lipofectamine 2000 (Thermo Fisher Scientific). Stable clones were obtained by selection using puromycin (0.8 µg/mL for LP6 cells and 0.6 µg/mL for LPS12 cells).

### Western blot analysis

Nuclear and cytoplasmic proteins were extracted using NE-PER™ Nuclear and Cytoplasmic Extraction Reagents (Thermo Fisher Scientific). Proteins were resolved using SDS-PAGE and transferred to an Immobilon-P nylon membrane (Merck Millipore, Billerica, MA). Each membrane was treated with BlockAce (Dainippon Pharmaceutical Co., Ltd., Suita, Japan) and reacted with a rabbit antibody against PPARγ (PP-A3409-00, 1:1000; Perseus Proteomics, Tokyo, Japan), a rabbit antibody against NUP98 (2598; 1:1000; Cell Signaling Technology, Danvers, MA), or a mouse antibody against TUBULIN (69-1251, 1:5000; ICN Biomedicals, Aurora, OH). After three cycles of 10-min washes in Tris-buffered saline supplemented with 0.5% Tween 20 (TBST), the blots were reacted with a secondary antibody conjugated with peroxidase (rabbit IgG, 7074, 1:1000; Cell Signaling Technology, mouse IgG, 7076, 1:1000; Cell Signaling Technology) and rewashed. Enhanced chemiluminescence detection was performed using an ECL kit (Cytiva, Marlborough, MA).

### Treatment of cells with 5-aza-dC and rosiglitazone

Cells were seeded in a 6-well plate on Day 0 in triplicate (8 × 10^4^ cells/well for LP6 and 1 × 10^5^ cells/well for LPS12) and were treated with 0.3 µM of 5-aza-2’-deoxycytidine (5-aza-dC, Sigma-Aldrich, St Louis, MO) on Days 1 and 3. On Days 5 and 6, 50 µM of rosiglitazone (Sigma-Aldrich) was added to the medium. On Day 7, the cells were analyzed for morphology viability and harvested for RNA extraction. The dose and treatment schedule of 5-aza-dC were based on previous reports (20, 26, 27). Total RNA was extracted using ReliaPrep RNA Miniprep Systems (Promega, Madison, WI).

### Analyses of cell morphology, cell viability, and Oil Red O staining

Cell morphology was analyzed under a phase-contrast microscope, and at least two images were captured at high magnification using a BZ-X710 microscope system (Keyence, Osaka, Japan). Cell viability was assessed using a TC10 automated cell counter (Bio-Rad Laboratories Inc., Hercules, CA). For Oil Red O staining, LP6 cells were fixed with 10% formaldehyde and incubated at 37°C for 15 min. After washing with 60% isopropanol, the samples were stained with 60% Oil Red O (Sigma-Aldrich) in isopropanol. The ratio of Oil Red O-positive lipid area to the total cell area was measured using the BZ-X (BZ-H4A) Analyzer software (six fields for mock-treated cells, and four fields for cells treated with 5-aza-dC and rosiglitazone).

### Unsupervised hierarchical clustering, principal component analysis, and correlation of DNA methylation profiles

Unsupervised hierarchical clustering was conducted using R 3.61 with the Heatplus package from Bioconductor. Principal component analysis and Spearman’s correlation analysis were performed using the prcomp() and cor() functions in R.

### Identification of regulatory regions and PPARγ binding sites in adipocytes

ChIP-seq data for H3K27ac in pre-adipocyte cells (hASCs) (https://www.ncbi.nlm.nih.gov/; GSM534468) (28) were downloaded and processed to categorize them into promoters, typical-enhancers, and super-enhancers using an algorithm described in a previous study (29). The read density was normalized to reads per million per base pair (rpm/bp), and all peaks were ranked along the x-axis on the total background-subtracted ChIP-seq density in increasing order. Enhancers were defined as any regions with H3K27ac peaks, excluding those within ±2000 bp of the transcription start site (TSS). Super-enhancers were distinguished from typical enhancers if their locations were above the point where the slope of the plot was 1. A gene was considered to be associated with an enhancer when its TSS was within 50 k bp of the enhancer. The ChIP-seq data for PPARγ in pre-adipocyte cells were downloaded (https://www.ncbi.nlm.nih.gov/; GSM534493 for PPARγ1 and GSM534494 for PPARγ2).

### Statistical analysis

Differences in β-values and gene expression were assessed using an unpaired Student’s *t*-test with significance set at *P* < 0.05 obtained from a two-sided test. The Jonckheere-Terpstra test was used to evaluate the trend of differences in β-values among normal adipose tissues, WDLPSs, and DDLPSs. Statistical significance was set at *P* < 0.01. All statistical analyses were performed using Excel (Microsoft Corp., Seattle, WA).

## Results

### DDLPSs showed heterogeneity in DNA methylation

**Figure 1:**
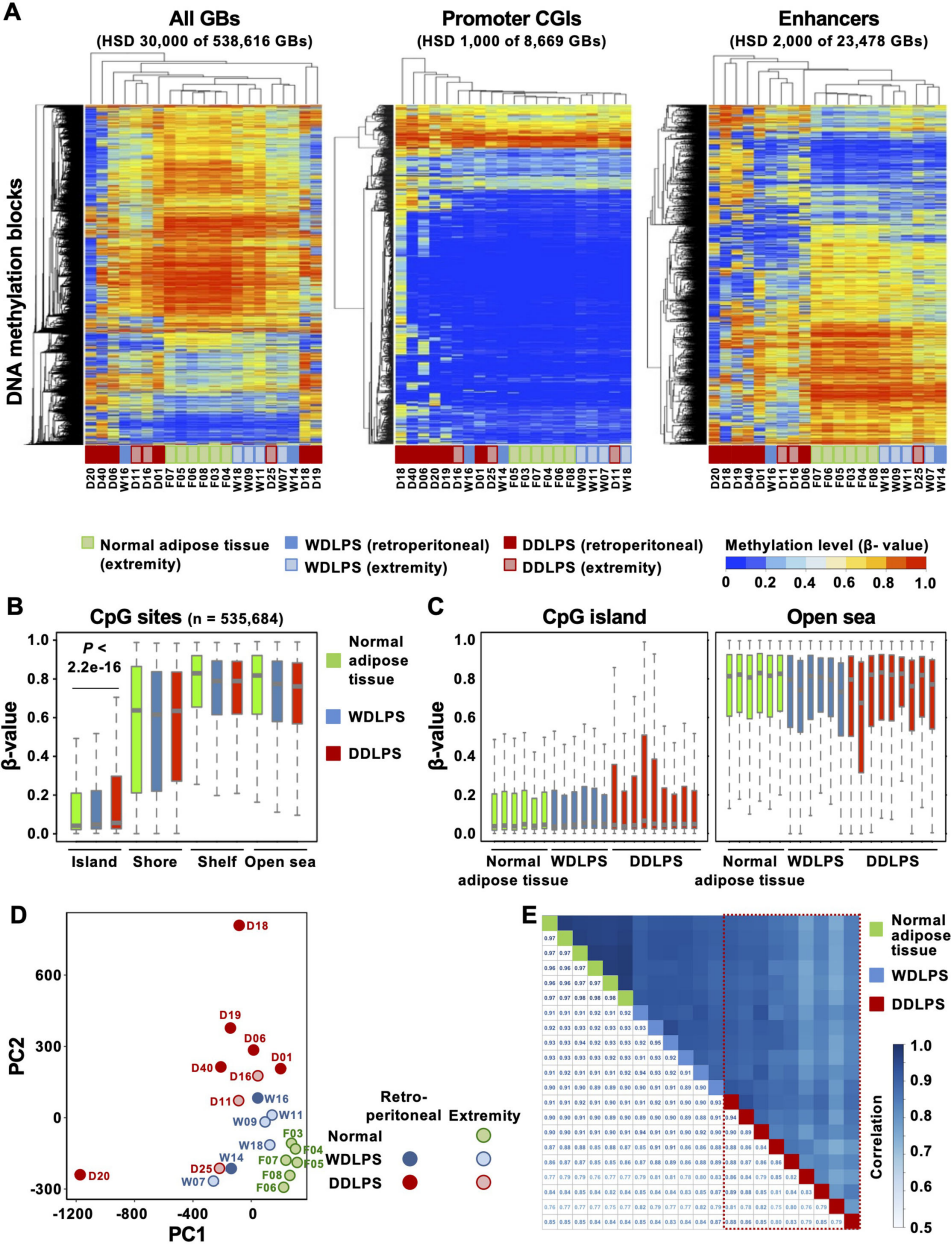
Genome-wide DNA methylation profiles of normal adipose tissues, well-differentiated liposarcomas (WDLPSs), and dedifferentiated liposarcomas (DDLPSs). **(A)** Hierarchical clustering using the genomic blocks (GBs) with the highest standard deviation (HSD). DNA methylation profiles at the enhancers classified the samples into three tissue types, whereas those at the promoters did not. **(B)** Box plots of β-values of CpG sites in specific regions against the CpG island (CGI). DDLPSs showed hypermethylation at CGIs, but not in the shores, shelves, or open seas. Results are shown as mean ± SE, and statistical significance was tested using an unpaired Student’s *t*-test. **(C)** Box plots of β-values of individual samples. DDLPSs showed intertumoral heterogeneity in their DNA methylation profiles. **(D)** Principal component analysis using all GBs. Normal adipose tissues were closely positioned, whereas DDLPSs were scattered. **(E)** Correlation analysis using all GBs. DDLPSs showed large variation in their degrees of correlation, whereas WDLPSs and normal adipose tissues showed high correlations.

Genome-wide DNA methylation profiles of normal adipose tissues (n = 6), WDLPSs (n = 6), and DDLPSs (n = 9) were evaluated using Infinium MethylationEPIC BeadChip. Hierarchical clustering analysis using 30,000 GBs with the highest standard deviation (HSD) revealed that normal adipose tissues and WDLPSs showed similar profiles, whereas the profiles of DDLPSs were different (**Figure 1A**). When promoter CpG islands (CGIs), defined by the annotations of TSS200 and CGI, were analyzed using 1,000 GBs with HSD, DDLPSs, especially those originating from the retroperitoneum, exhibited higher methylation levels than did normal adipose tissues and WDLPSs (**Figure 1A**). When enhancers were analyzed using 2,000 GBs with HSD, normal adipose tissues and WDLPSs again showed similar profiles, but the former had more hypermethylated GBs. DDLPSs had very different profiles and showed both hyper- and hypomethylation (**Figure 1A**).

Next, to analyze the characteristics of the genomic regions that were differentially methylated in the three groups, methylation differences were analyzed for 535,684 CpG sites based on their positions against a CGI. Those in CGIs were hypermethylated in DDLPSs, but those in the shore, shelf, and open sea were not (**Figure 1B**). When individual samples were analyzed, the four DDLPSs displayed higher methylation levels in CGIs than in normal adipose tissues and WDLPSs (**Figure 1C**). In the open sea, one DDLPS showed lower methylation levels than in WDLPSs and normal adipose tissues. This suggested the presence of intertumoral heterogeneity in DNA methylation profiles of DDLPSs.

To pursue the intertumoral heterogeneity in DNA methylation profiles of DDLPSs, we conducted principal component analysis and correlation analysis using all the samples. Principal component analysis also showed that normal adipose tissues were closely positioned, whereas DDLPSs were scattered (**Figure 1D**). The correlation coefficients between two of the 9 DDLPSs showed a large variation from 0.72 to 0.95 (marked by a trapezoid with a red broken line), whereas those of the 6 WDLPSs and 6 normal adipose tissues consistently had large values (0.93 to 0.96 for WDLPSs and 0.98 to 0.99 for normal adipose tissues) (**Figure 1E**). The influence of stromal cell contamination on DNA methylation levels was assessed by estimating tumor cell content. Since DDLPSs exhibited higher tumor cell contents than WDLPSs (**Supplementary Figures S1A, B**), stromal cell contents could not account for the large variation of DNA methylation levels in DDLPSs. In addition, cases D18 and D20, outliers in PCA (**Figure 1D**), had high tumor cell contents, excluding the influence of stromal cell contamination on the strong variation of DNA methylation levels. Together with the data from the hierarchical cluster analysis, intertumoral heterogeneity in DNA methylation was observed in DDLPSs.

### Genomic regions hypermethylated in DDLPSs involved adipogenic genes

**Figure 2:**
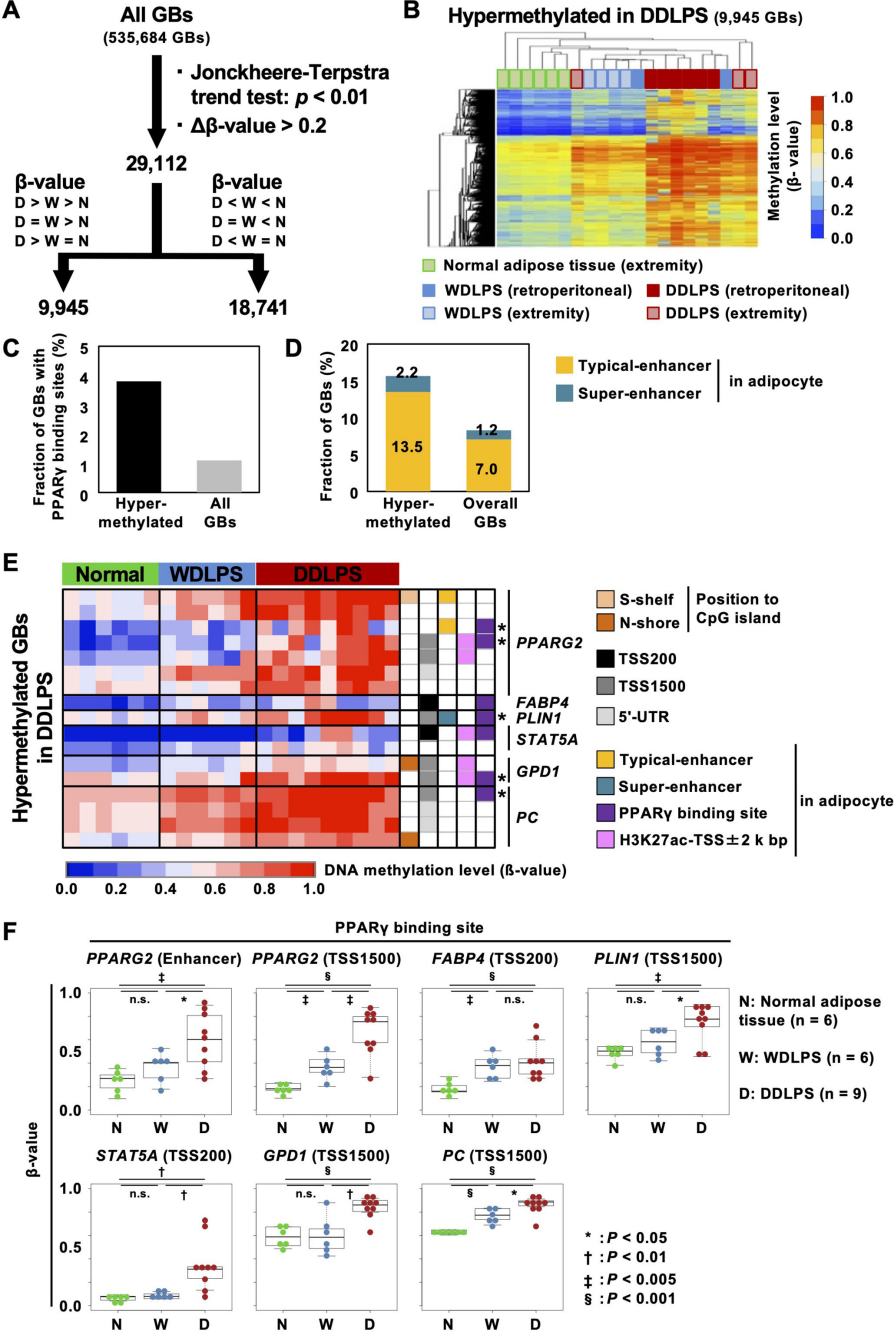
Characteristics of GBs hypermethylated in DDLPSs. **(A)** Identification of differentially methylated regions in DDLPSs using the Jonckheere-Terpstra trend test. A total of 29,112 GBs were identified as GBs significantly different among the three groups. **(B)** Heatmap of GBs hypermethylated in DDLPSs. A total of 9,945 GBs were hypermethylated in DDLPSs. **(C)** Fraction of GBs with PPARγ binding sites. The GBs hypermethylated in DDLPSs had a high chance of containing a PPARγ binding site. **(D)** Fraction of GBs overlapping typical and super-enhancers in adipocytes. The GBs hypermethylated in DDLPSs were enriched in super-enhancers in adipocytes. **(E)** DNA methylation statuses of the downstream genes of PPARγ. Asterisks indicate PPARγ binding sites heavily methylated in DDLPSs. The regulatory regions of PPARγ target genes, such as *PPARG*, *FABP4*, *PLIN1*, *STAT5A*, *GPD1*, and *PC*, were methylated in DDLPSs. **(F)** DNA methylation levels of PPARγ binding sites. DNA methylation levels of PPARγ binding sites in DDLPSs were significantly increased compared with those of normal adipose tissues and WDLPSs.

To explore the features of genomic regions methylated in DDLPSs, GBs with increasing or decreasing β-values in the order of normal adipose tissues, WDLPS, and DDLPS were isolated using the Jonckheere-Terpstra trend test **Figure 2A**). Among all GBs (n = 535,684), 9,945 GBs were hypermethylated in DDLPSs with Δβ-values between those of DDLPSs and normal adipose tissues (*P* < 0.01), and 18,791 GBs were hypomethylated (**Figures 2A, B**). Compared to the overall GBs, the GBs hypermethylated in DDLPSs were enriched at 5’ UTR and in the CGI shore (**Supplementary Figures S2A, B**) with a high fraction having a binding site of PPARγ, a master transcription factor for adipogenic differentiation (30) (**Figure 2C**). GBs hypomethylated in DDLPSs were enriched in the intergenic regions and were excluded from CGIs. Enrichment analysis using ChIP-Atlas showed that GBs hypermethylated in DDLPSs significantly overlapped the binding sites of transcription regulators, such as CTCF, SMARCC1, PPARγ, JUN, and MED1 (**Supplementary Figure S2C**), whereas hypomethylated GBs did not show significant overlap.

Among the hypermethylated binding sites, the presence of a binding site of PPARγ suggested a possibility that genes involved in adipogenesis were dysregulated by enhancer hypermethylation in DDLPSs and that this led to a disturbance in adipogenic differentiation. To explore this possibility, we first isolated GBs marked with histone H3K27 acetylation in adipocytes based on a previous report (28) and classified them into typical and super-enhancers (**Supplementary Figure S2D**). Compared to the overall GBs, the GBs hypermethylated in DDLPSs frequently overlapped with the typical and super-enhancer GBs (**Figure 2D**).

When downstream genes of PPARγ, such as *PPARG, FABP4*, *PLIN1*, *STAT5A*, *GPD1*, and *PC*, were analyzed, *PPARG2* enhancers, upstream regions of *GPD1* and *PC* (TSS1500), and *PLIN1* super-enhancer with PPARγ binding sites were highly methylated in DDLPSs (shown with asterisks in **Figure 2E**, **Supplementary Figure S3**). In addition, the *FABP4* promoter with a PPARγ binding site was also methylated in DDLPSs. A significant increase in DNA methylation levels of PPARγ binding sites in DDLPSs compared to normal adipose tissues and WDLPSs was confirmed by statistical analysis (**Figure 2F**). We examined the DNA methylation levels of additional genes involved in adipogenesis, namely *SREBF1*, *CEBPA*, *CEBPB*, *CFD*, and *LPL*, and found that the upstream regions of *SREBF1* and *CFD* exhibited higher methylation levels in DDLPSs than in normal adipose tissues and WDLPSs (**Supplementary Figure S6A**). Importantly, aberrant methylation of PPARγ downstream genes in DDLPSs was also observed in an independent cohort from the TCGS-SARC database (**Supplementary Figure S4**).

### Hypermethylated adipogenic genes were downregulated in DDLPSs

**Figure 3:**
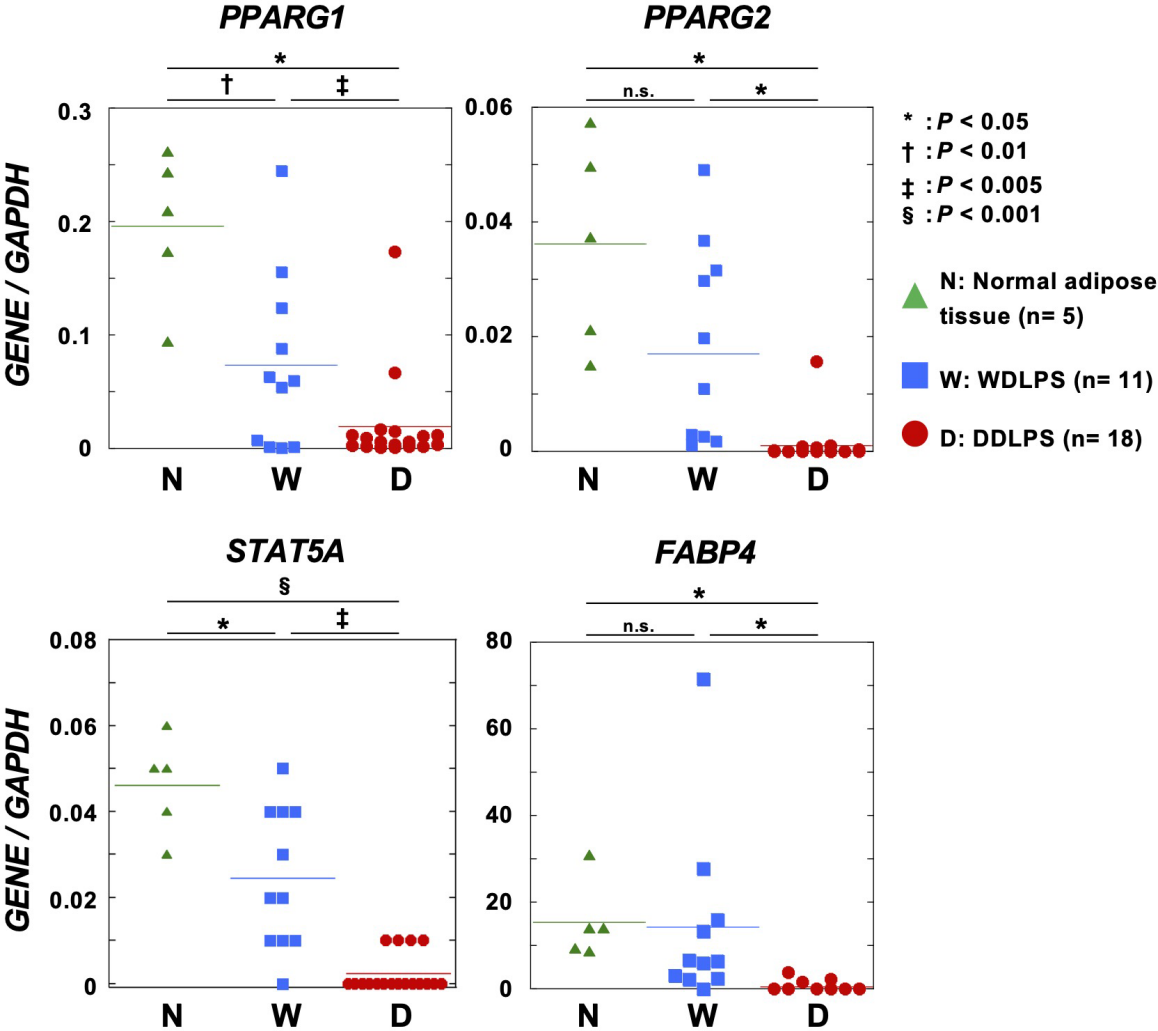
Expression levels of adipogenic genes in normal adipose tissues, primary WDLPSs, and DDLPSs. *STAT5A*, *FABP4*, and two transcript variants of *PPARG* were downregulated in primary DDLPSs. Statistical significance was tested using an unpaired Student’s *t*-test. *P < 0.05, ^†^P < 0.01, ^‡^P < 0.005, ^§^P < 0.001.

To analyze the impact of DNA methylation on gene expression, the expression of adipogenic genes in normal adipose tissues (n = 5), WDLPSs (n = 11), and DDLPSs (n = 18) was analyzed using RT-qPCR. One transcript variant of *PPARG*, *PPARG1* known to be expressed in nearly all cells, was highly expressed in normal adipose tissues and in approximately 60% of WDLPSs. In contrast, only 10% of DDLPSs expressed *PPARG1*, and the remaining DDLPSs had downregulation of *PPARG1*. The *PPARG1* promoter itself was not methylated in any of the nine DDLPSs (data not shown). The other transcript variant of *PPARG*, *PPARG2*, whose expression is restricted to adipose tissue, was expressed in normal adipose tissues and in a half of the WDLPSs, but downregulated in most of DDLPS (**Figure 3**). The proximal enhancers of *PPARG2* (shown by * in **Figure 2E**) were methylated (β-value ≥ 0.6) in five of nine DDLPSs, but in none of six WDLPSs and six normal adipose tissues (**Figure 2E**). Two PPARγ target genes, *STAT5A* and *FABP4*, were expressed in normal adipose tissues and WDLPSs but downregulated in DDLPSs (**Figure 3**). Protein downregulation of FABP4 in DDLPS was previously reported (31). Among the DDLPSs, those with complete loss of *STAT5A* expression had high DNA methylation levels, suggesting the importance of DNA methylation of its promoter.

### DNA demethylating agent enhanced adipose differentiation together with PPARγ2

The PPARγ agonist is known to induce differentiation of liposarcoma to adipose tissues (32, 33), but a phase II trial of rosiglitazone against liposarcoma showed no clinical response (34). In the present study, based on the presence of aberrant DNA methylation of genes involved in adipogenesis, we hypothesized that when combined with a DNA demethylating agent, rosiglitazone may be effective in inducing DDLPS differentiation.

**Figure 4:**
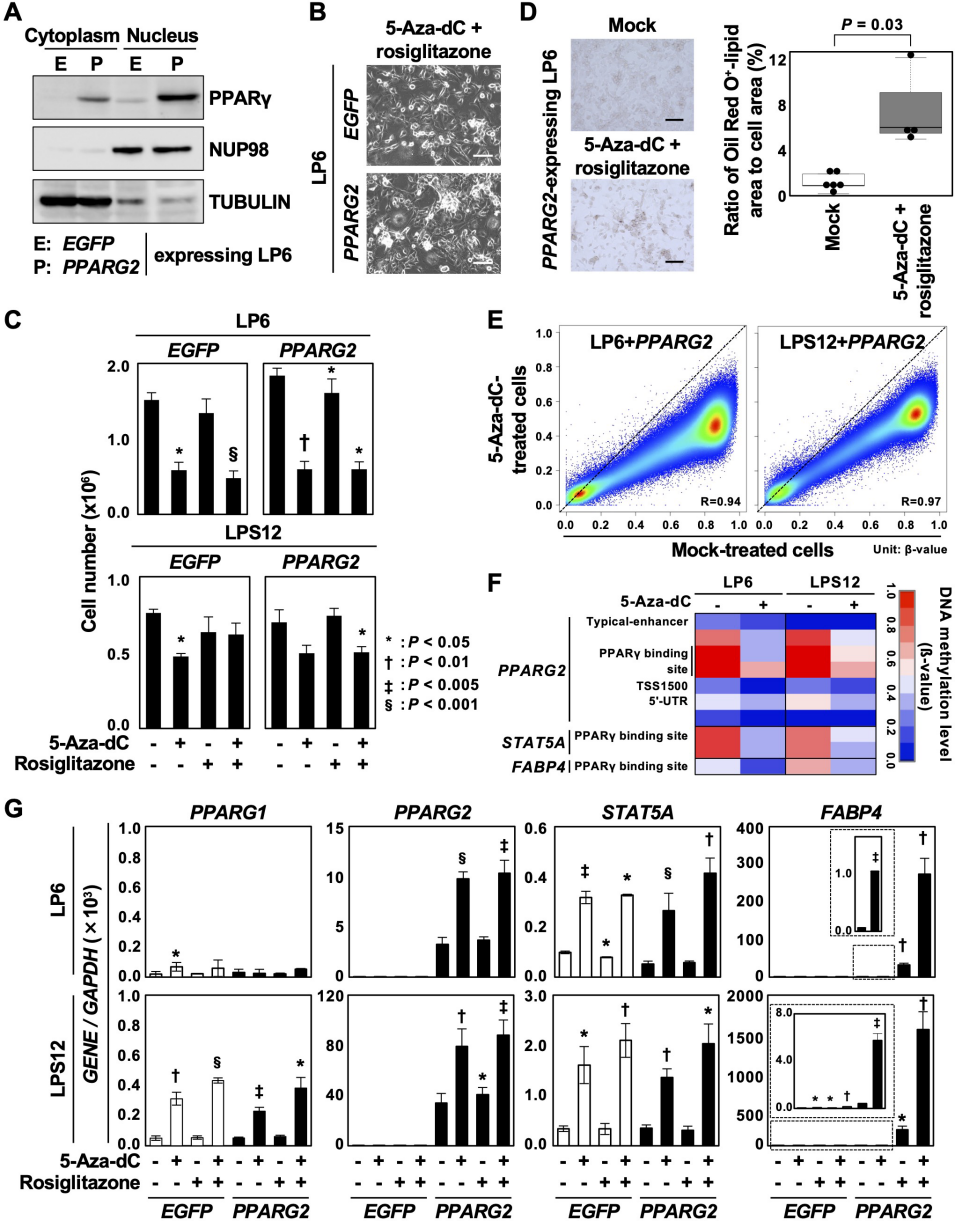
Enhancement of adipose differentiation by the combined treatment with 5-aza-dC and a PPARγ agonist. **(A)** PPARγ2 protein expression in LP6 cells with *PPARG2* overexpression. Overexpression of the PPARγ2 protein was confirmed. **(B)** Representative cell morphology. No clear difference in cell morphology was observed between *EGFP*- and *PPARG2*-expressing LP6 cells after treatment with 5-aza-dC and rosiglitazone. Scale bar = 100 µm. **(C)** Changes in cell numbers after the treatment with 5-aza-dC and rosiglitazone. Cell number was decreased by the treatment with 5-aza-dC in both *EGFP*- and *PPARG2*-expressing LP6 and LPS12 cells. Data are shown as the mean ± SD obtained from three experiments. **(D)** Representative Oil Red O staining. Oil Red O-positive cells slightly increased in *PPARG2*-expressing LP6 cells treated with 5-aza-dC and rosiglitazone (left panel). The ratio of Oil Red O-positive lipid area to the total cell area was quantified (right panel). Statistical significance was tested using an unpaired Student’s *t*-test. Scale bar = 100 µm. **(E)** Genome-wide DNA demethylation by 5-aza-dC in LP6 and LPS12 cells expressing PPARG2. Genome-wide DNA methylation levels were analyzed using the Infinium MethylationEPIC BeadChip in mock-treated cells and 5-aza-dC-treated cells (0.3 µM) and compared. Methylation levels are shown as β-values (0.0 to 1.0). Comparison between mock-treated cells and treated cells showed that 5-aza-dC induced DNA demethylation of virtually all genomic blocks. **(F)** DNA methylation status of the downstream genes of PPARγ. The regulatory regions of *PPARG2*, *STAT5A*, and *FABP4* were demethylated by 5-aza-dC treatment. **(G)** Expression levels of the genes methylated in DDLPSs after the treatment with 5-aza-dC and rosiglitazone. Treatment with 5-Aza-dC additively induced the expressions of *PPARG2* and *STAT5A* in *PPAR2*-expressing LP6 and LPS12 cells. The expression of *FABP4* was synergistically induced by the combined treatment. Each RT-qPCR analysis was performed in triplicate, and the results are presented as mean ± SE. *P < 0.05, ^†^P < 0.01, ^‡^P < 0.005, ^§^P < 0.001.

As *PPARG* itself was downregulated in DDLPSs (**Figure 3**), we first introduced the *PPARG2* transcript isoform into LP6 and LSP12 cells and confirmed sufficient expression of the PPARγ2 protein (**Figure 4A** for LP6). To investigate the enhancing effects of 5-aza-2’-deoxycytidine (5-aza-dC) on the rosiglitazone-induced differentiation, LP6 and LPS12 cells expressing *EGFP* or *PPARG2* were first treated with 0.3 µM of 5-aza-dC for 3 d, then treated with 50 µM of rosiglitazone for an additional 2 d (**Supplementary Figure S5A**). Differences in cell morphology were unclear between *EGFP*- and *PPARG2*-expressing LP6 and LPS12 cells when 5-aza-dC and rosiglitazone were added (**Figure 4B**, **Supplementary Figure S5B**). Treatment with 5-aza-dC decreased the number of both *EGFP*- and *PPARG2*-expressing LP6 and LPS12 cells, showing a repressive effect of 5-aza-dC on cell growth (**Figure 4C**). An increase in Oil Red O-positive cells was observed among *PPARG2*-expressing LP6 cells treated with 5-aza-dC and rosiglitazone (**Figure 4D**).

The demethylation effect of 5-aza-dC was analyzed in LP6 and LPS12 expressing *PPARG2* using the Infinium MethylationEPIC BeadChip. Compared with the mock-treated cells, the 5-aza-dC-treated cells showed decreased β-values at virtually all CpG sites in both cell lines, meaning that the demethylation ability of 5-aza-dC was achieved by the dose and treatment schedule used in this study (**Figure 4E**). In addition, the demethylation occurred at the regulatory regions of *PPARG2*, *STAT5A*, and *FABP4* in the 5-aza-dC-treated cells (**Figure 4F**). 5-Aza-dC treatment also induced demethylation at the regulatory regions of *CEBPA, CEBPB, CFD* and *LPL* among the genes involved in adipogenesis (**Supplementary Figure S6B**). These data indicate that 5-aza-dC treatment reprogrammed adipogenic genes and enhanced the differentiation induction by rosiglitazone.

The expression of *PPARG1*, whose promoter was not methylated in DDLPS, was induced by 5-aza-dC treatment in LPS12 cells, potentially due to the upregulation of its transcription factors. *PPARG2* and *STAT5A* were additively induced by 5-aza-dC treatment in *PPAR2*-expressing LP6 and LPS12 cells, although rosiglitazone alone could not induce their expressions (**Figure 4G**), which may be attributable to the high methylation status of PPARγ binding sites within the regulatory regions of these genes. The combination of 5-aza-dC and rosiglitazone synergistically induced the expression of *FABP4* in *PPARG2*-expressing LP6 and LPS12 cells, whereas monotherapy with 5-aza-dC induced only a slight expression (**Figure 4G**). These data indicated that DNA demethylation by 5-aza-dC prior to rosiglitazone treatment may be effective, at least in specific contexts.

## Discussion

This study shows that aberrant DNA methylation of adipogenic gene enhancers is involved in the pathogenesis of DDLPS. The genomic regions hypermethylated in DDLPSs had a high fraction of adipocyte enhancers and were associated with the repression of PPARγ target genes, such as *STAT5A* and *FABP4*. Aberrant hypermethylation at the regulatory regions of *PPARG2*, *SATA5A*, and *FABP4* was reversed by treatment with a DNA demethylating agent in DDLSP cell lines. Although single treatment with a PPARγ agonist led only to limited upregulation of these genes, the prior treatment with a DNA demethylating agent had a synergistic effect on the expression of *FABP4*, which was expected to lead to enhanced DDLPS differentiation. These results indicated a potential therapeutic value of targeting aberrant DNA methylation, along with activation of PPARγ, in DDLPS.

The GBs hypermethylated in DDLPSs were enriched with enhancers in adipocytes, suggesting that enhancer methylation is a characteristic of DDLPS. Enhancers are known to have low DNA methylation level and be sensitive to alterations in DNA methylation during differentiation or development (35–37). One possible mechanism for maintaining low methylation in enhancers is cooperative binding of multiple transcription factors that block *de novo* methylation and/or increase active demethylation by TET enzymes (37–39). If the regulation of transcription factors is impaired, enhancers are critically involved in disturbances in differentiation because of their importance in determining cell fate (17–19, 40). *PPARG1* and *PPARG2* expression was lower in DDLPSs than in normal adipose tissues and WDLPSs, and this may have led to methylation of adipocyte enhancers with PPARγ binding sites, making simple *PPARG2* overexpression ineffective for inducing differentiation. Taken together, the downregulation of PPARγ expression by some mechanism appears to have led to the methylation of enhancers that resulted in irreversible loss of differentiation. The presence of enhancer methylation in DDLPSs can explain the difference in differentiation induction by ectopic expression of PPARγ in human fibroblasts (32) and the lack of differentiation induction in DDLPS cells.

The limitations of this study lie in the relatively small sample size, the age differences among the groups, and the lack of our own validation cohort. On the other hand, since aberrant DNA methylation of PPARγ downstream genes was also observed in an independent cohort in the TCGA database, we assume that the aberrant methylation associated with DDLPS development could be generalized. Additionally, a small number of genes showed synergistic responses to the treatment with a DNA demethylating agent and rosiglitazone. We believe that, if transcriptome analysis is conducted, more genes will be identified.

In conclusion, aberrant DNA methylation of adipogenic gene enhancers occurred in DDLPSs, and targeting aberrant DNA methylation before the induction of differentiation by PPARγ agonists appears to be a promising strategy.

## Data Availability

The datasets presented in this study can be found in online repositories. The names of the repository/repositories and accession number(s) can be found below: https://www.ncbi.nlm.nih.gov/, GSE252182 https://www.ncbi.nlm.nih.gov/, GSE278425.
