# Supplementary material for: Epigenetic disruption of adipogenic gene enhancers in dedifferentiated liposarcomas and its therapeutic value
Source: Front Oncol. 2025 Apr 30;15:1419877. doi: 10.3389/fonc.2025.1419877 (PMC12074953; doi:10.3389/fonc.2025.1419877)
Supplement: Supplementary file 1 [file DataSheet1.pdf]

Supplementary Materials

Supplementary Data

Table S1. Clinical and pathological information of WDLPS and DDLPS cases analyzed in this study

	Code No.	Age (years)	Sex*	Tumor site	MDM2/CDK4 Amplification§	Amplification of other genes§	Mutation§
DDLPS	D11	55	M	Extremity	Yes		
	D16	62	M	Extremity	Yes	*DDR2*	
	D25	55	M	Extremity	Yes	*IGF1R*	
	D01	52	F	Retroperitoneal	Yes	*ROS1*	
	D06	74	M	Retroperitoneal	Yes	*RAC1, MYC, CRKL*	
	D18	74	M	Retroperitoneal	Yes		
	D19	63	M	Retroperitoneal	Yes		
	D20	64	M	Retroperitoneal	Yes		*ARID1A*
	D40	76	F	Retroperitoneal	Yes	*ROS1, CD274, EP300*	*FGFR1*
WDLPS	W07	30	M	Extremity	Yes	*MDM4*	
	W09	44	M	Extremity	Yes	*ERBB3*	
	W11	47	M	Extremity	Yes		
	W18	81	M	Extremity	Yes	*DDR2*	
	W14	60	F	Retroperitoneal	Yes	*NTRK1*	
	W16	59	F	Retroperitoneal	Yes	*IDH1*	
Normal adipose tissue	F03	40	F	Extremity			
	F05	44	M	Extremity			
	F04	71	F	Extremity			
	F06	66	F	Extremity			
	F07	43	F	Extremity			
	F08	74	M	Extremity			

*M: Male, F: Female

§Reference: Asano N. et al. Oncotarget. 2017;8(8):12941-52

Table S2. Primers for RT-qPCR

Gene symbol	Sequence (forward)	Sequence (reverse)	Annealing temp. (˚C)
GAPDH	AGGTGAAGGTCGGAGTCAACG	AGGGGTCATTGATGGCAACA	58
PPARG1	AGAAGCCAACACTAAACCACAAA	CAGAATGGCATCTCTGTGTCAAC	58
PPARG2	CTGTCTGCAAACATATCACAAG	GGAGTGGTCTTCCATTACGG	57
STAT5	CCTTCTTGTTGCGCTTTAGTG	ATGGTTTCAGGTTCCACAGG	58
FABP4	TGCAGAAATGGGATGGAAAATCA	CATAAACTCTCGTGGAAGTGACG	58

Supplementary Figures

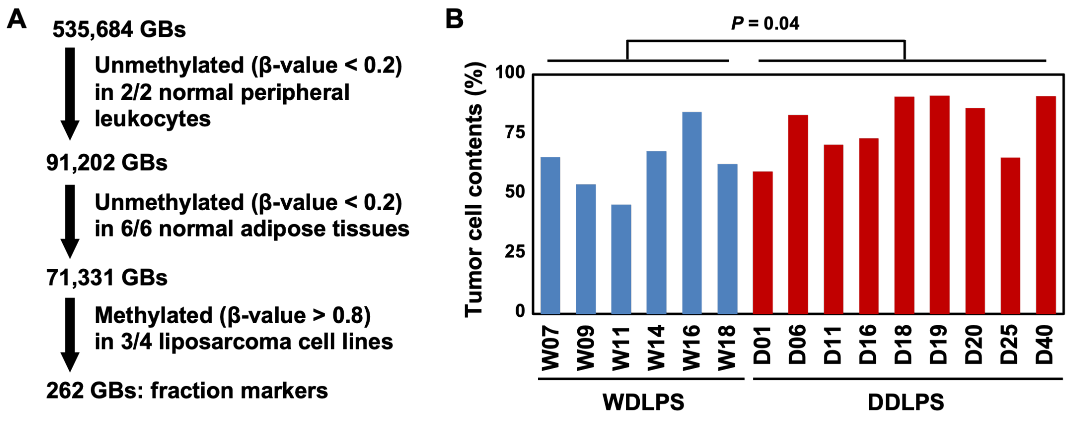

Figure S1

Tumor cell contents in liposarcomas. (A) Isolation of the fraction markers. Tumor cell contents were estimated using DNA methylation statuses of 262 genomic blocks (GBs) unmethylated (β-value < 0.2) in normal peripheral leukocytes and normal adipose tissues but methylated (β-value > 0.8) in three or more of four liposarcoma cell lines. (B) Tumor cell contents of WDLPSs and DDLPSs. DDLPSs exhibited higher tumor cell contents than WDLPSs. Statistical significance was tested using an unpaired Student's *t*-test.

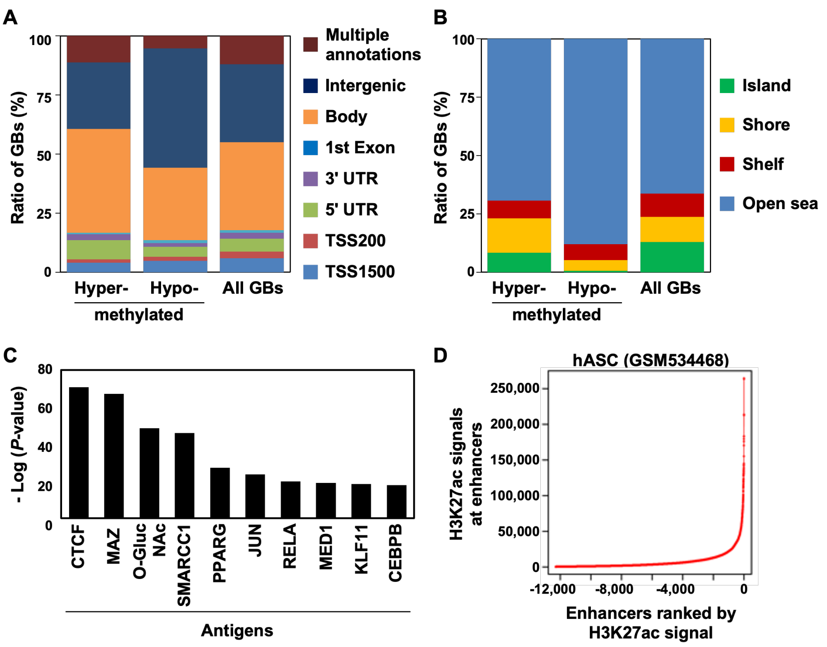

Figure S2

Genomic features of differentially methylated GBs. (A) Positions of differentially methylated GBs against a gene. GBs hypermethylated in DDLPSs were enriched at 5' UTR and the gene body, whereas GBs hypomethylated in DDLPSs were in the intergenic regions. (B) Positions of differentially methylated GBs against a CpG island. GBs hypermethylated and hypomethylated in DDLPSs were enriched at the shore of CGIs and the open sea, respectively. (C) Enrichment analysis using ChIP-Atlas. GBs hypermethylated in DDLPSs significantly overlapped the binding regions of transcription regulators, including *PPARG*. (D) Isolation of super-enhancers in adipocyte. Typical and super-enhancers in adipocytes were identified based on the aggregated intensity of histone H3K27 acetylation.

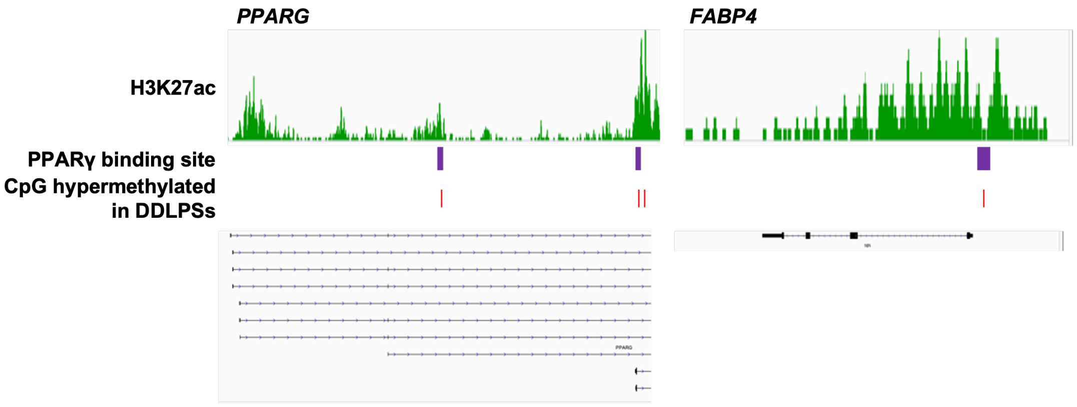

Figure S3

Relationship among H3K27 acetylation peaks and PPARγ binding sites in adipocytes, and CpG sites hypermethylated in DDLPSs. At the loci of *PPARG2* and *FABP4*, the PPARγ binding sites with H3K27ac in adipocytes were hypermethylated in DDLPSs.

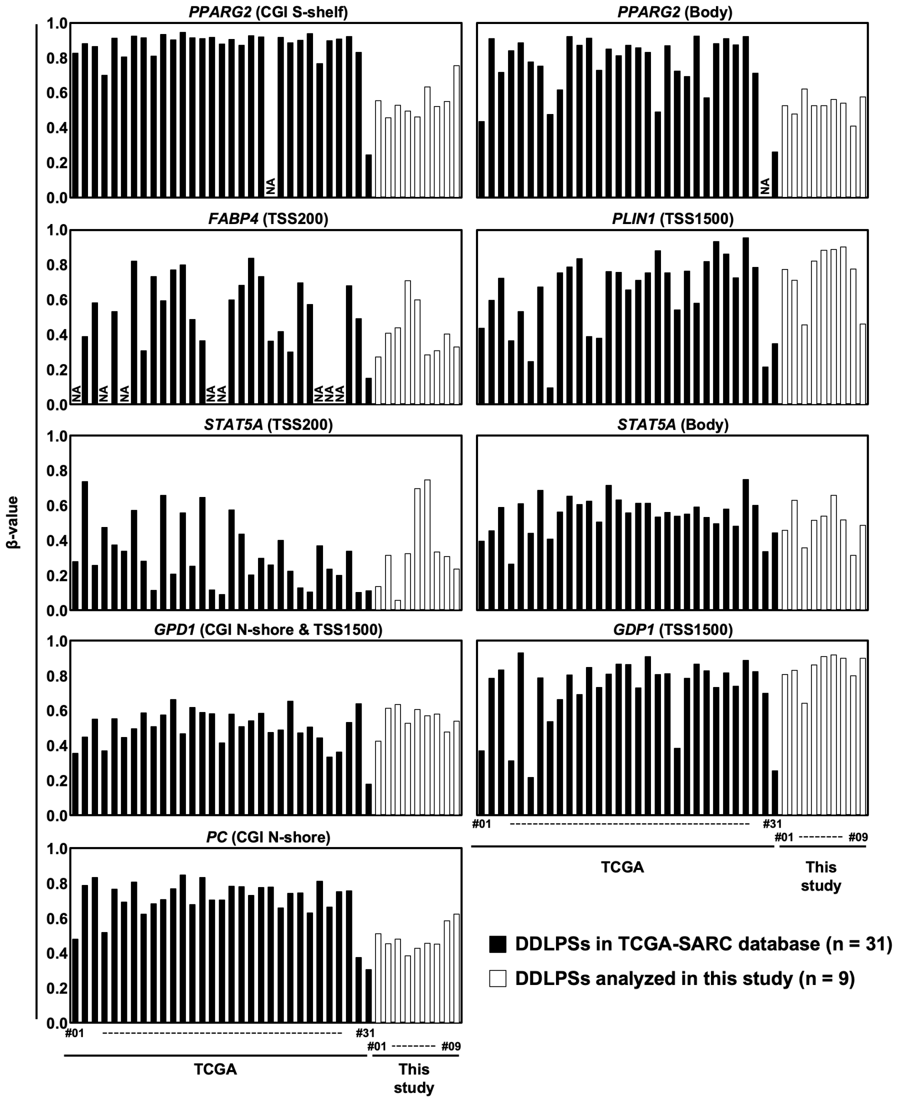

Figure S4

Validation of aberrant DNA methylation of PPARγ downstream genes in DDLPSs. DNA methylation profiles of 31 DDLPSs were obtained from the TCGA-SARC database. Focusing on probes common to both Infinium EPIC and 450K arrays, consistent DNA methylation of *PPARG2*, *FABP4*, *PLIN1*, *GPD1*, and PC was observed in an independent cohort from the TCGS-SARC database.

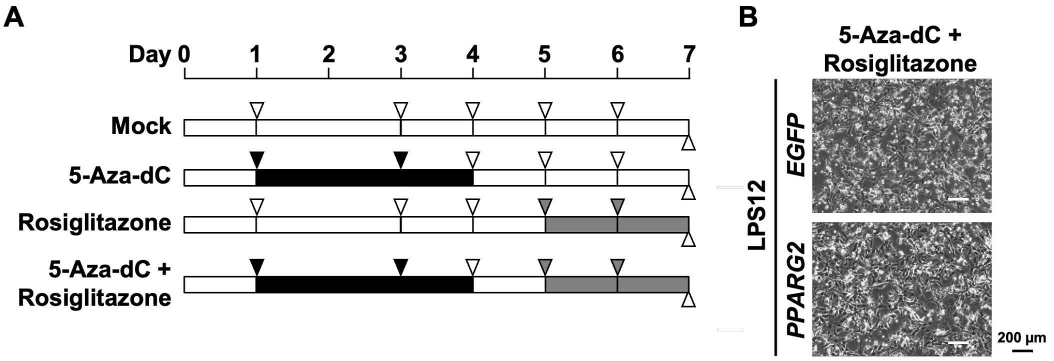

Figure S5

Treatment of DDLPS cell lines with 5-aza-dC and a PPARγ agonist. (A) Schedule of the combined treatment of 5-aza-dC (0.3 µM) and rosiglitazone (50 µM). (B) Representative cell morphology. No clear difference in cell morphology was observed between *EGFP*- and *PPARG2*-expressing LPS12 cells after treatment with 5-aza-dC and rosiglitazone. Scale bar = 200 µm.

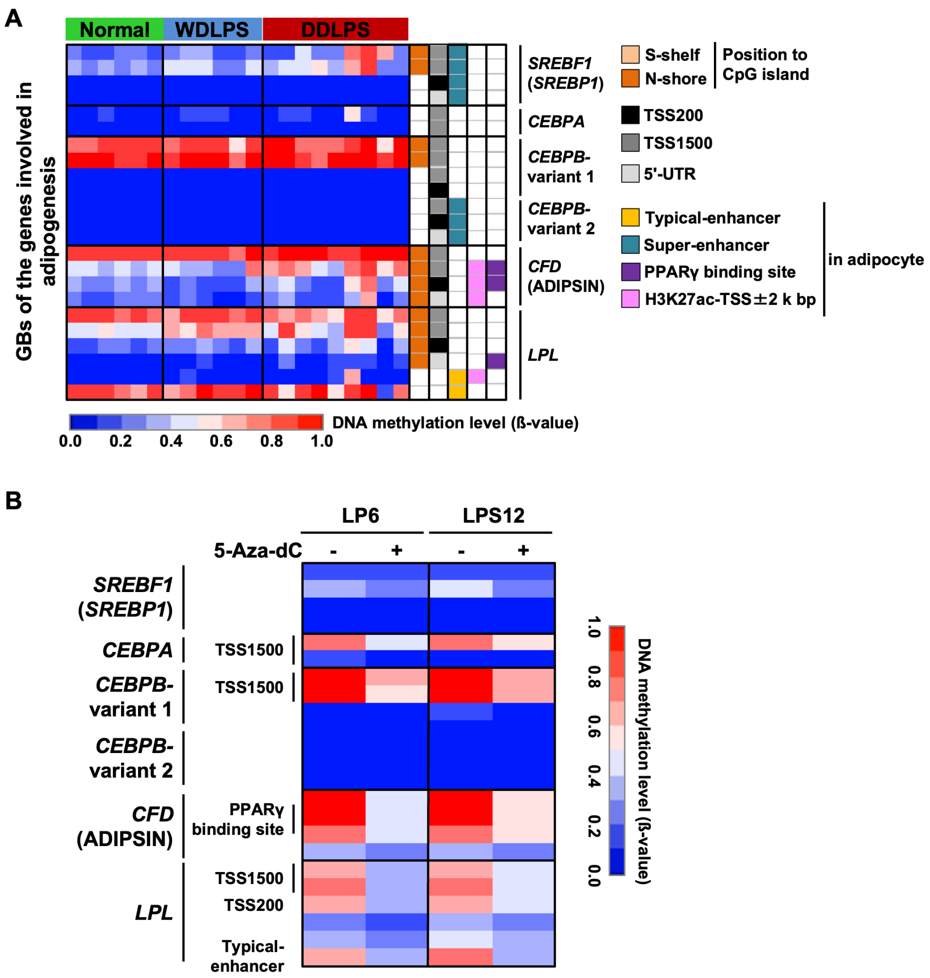

Figure S6

DNA methylation statuses of the genes involved in adipogenesis. (A) DNA methylation levels of the genes involved in adipogenesis in normal adipose tissues, WDLPSs, and DDLPSs. The regulatory regions of *SREBF1* and *CFD* were more methylated in DDLPSs. (B) DNA methylation levels of the genes involved in adipogenesis in LP6 and LPS12 cells. The regulatory regions of *CEBPA*, *CEBPB*, *CFD*, and *LPL* were demethylated by 5-aza-dC treatment.
